# MicroRNA Expression Profiling in Mild Asthmatic Human Airways and Effect of Corticosteroid Therapy

**DOI:** 10.1371/journal.pone.0005889

**Published:** 2009-06-12

**Authors:** Andrew E. Williams, Hanna Larner-Svensson, Mark M. Perry, Gaynor A. Campbell, Sarah E. Herrick, Ian M. Adcock, Jonas S. Erjefalt, Kian Fan Chung, Mark A. Lindsay

**Affiliations:** 1 Biopharmaceutics Research Group, National Heart and Lung Institute, Imperial College, London, United Kingdom; 2 Airways Disease, National Heart and Lung Institute, Imperial College, London, United Kingdom; 3 Respiratory Translational Research Facility, Wythenshawe Hospital, School of Translational Sciences, University of Manchester, Manchester, United Kingdom; 4 Department of Experimental Medical Science, Division of Vascular and Airway Research, Lund University, Lund, Sweden; Helmholtz Zentrum München/Ludwig-Maximilians-University Munich, Germany

## Abstract

**Background:**

Asthma is a common disease characterised by reversible airflow obstruction, bronchial hyperresponsiveness and chronic inflammation, which is commonly treated using corticosteroids such as budesonide. MicroRNAs (miRNAs) are a recently identified family of non-protein encoding genes that regulate protein translation by a mechanism entitled RNA interference. Previous studies have shown lung-specific miRNA expression profiles, although their importance in regulating gene expression is unresolved. We determined whether miRNA expression was differentially expressed in mild asthma and the effect of corticosteroid treatment.

**Methodology/Principal Findings:**

We have examined changes in miRNA using a highly sensitive RT-PCR based approach to measure the expression of 227 miRNAs in airway biopsies obtained from normal and mild asthmatic patients. We have also determined whether the anti-inflammatory action of corticosteroids are mediated through miRNAs by determining the profile of miRNA expression in mild asthmatics, before and following 1 month twice daily treatment with inhaled budesonide. Furthermore, we have analysed the expression of miRNAs from individual cell populations from the airway and lung.

We found no significant difference in the expression of 227 miRNAs in the airway biopsies obtained from normal and mild asthmatic patients. In addition, despite improved lung function, we found no significant difference in the miRNA expression following one month treatment with the corticosteroid, budesonide. However, analysis of bronchial and alveolar epithelial cells, airway smooth muscle cells, alveolar macrophages and lung fibroblasts demonstrate a miRNA expression profile that is specific to individual cell types and demonstrates the complex cellular heterogeneity within whole tissue samples.

**Conclusions:**

Changes in miRNA expression do not appear to be involved in the development of a mild asthmatic phenotype or in the anti-inflammatory action of the corticosteroid budesonide.

## Introduction

The regulation of gene expression is important in maintaining tissue homeostasis, preventing inflammatory processes and cancer. Recently, microRNAs (miRNAs) have been shown to regulate gene expression by inhibiting protein translation. MiRNAs are a family of small, non-coding RNA molecules that are transcribed in the nucleus into complex hairpin-loop primary-miRNAs [1]. These are processed by the RNAse III-type enzyme Drosha [2], into precursor-miRNAs, which are transported into the cytoplasm by the exportin 5 complex [3]. Further processing by the enzyme dicer results in short double-stranded miRNAs approximately 22 nt in length [4]. One strand of the miRNA, referred to as the mature miRNA (the other strand miRNA^*^ is generally degraded), is incorporated into a large ribonucleoprotein (RNP) complex called the miRNA-induced silencing complex (miRISC), where it binds to its target mRNA [5], [6]. The miRNA:mRNA interaction occurs within the 3′-UTR of the target mRNA at specific miRNA-recognition elements (MREs), usually in a partially complementary manner [7], [8]. The result of miRNA binding in the miRISC is the inhibition of protein translation, either through inhibition of translation initiation/elongation or through mRNA destabilisation [9].

Several human diseases have now been associated with disregulated miRNA expression. For example, cardiac hypertrophy has been linked to an absence of miR-1, while cardiomyocyte differentiation is regulated by miR-1 through interaction with serum response factor [10]. Cardiac arrhymogenesis has also been linked to miR-1, as well as miR-133, both acting through the regulation of essential ion channel proteins [11]. Metabolic diseases have been linked to certain miRNAs including the control of insulin secretion by miR-375 [12] and lipid metabolism in the liver by miR-122 [13]. Inflammatory diseases are also likely to be regulated by miRNAs, as miR-203 and miR-146a are associated with psoriasis [14], while miR-155 regulates lung remodelling [15]. Both miR-146a and miR-155 have also been implicated in the development of rheumatoid arthritis, possibly by regulating components of the inflammatory response [16], [17]. Furthermore, the altered expression of miRNAs has also been associated with the progression of several cancers, including solid tumours and leukaemias. For example, overexpression of the miR-17-92 cluster is thought to influence tumour progression [18], and is controlled by the transcription factor c-myc [19]. Burkitt's lymphoma, Hodgkin's lymphoma and lung cancer have all been associated with miR-155 overexpression [20]–[22], while B cell chronic lymphocytic leukaemia has been linked to a deletion in the miR-15/miR-16 locus [23]. More recently, miR-34a has been demonstrated to be an important component of the p53 network, probably functioning to reinforce the tumour suppressor effects of p53 [24], [25]. However it is unclear what influence miRNAs have over other human diseases such as asthma.

Asthma is a chronic inflammatory condition of the airways that is characterised by airway hyperresponsiveness and intermittent episodes of wheezing, chest tightness and shortness of breath. It is usually classified according to its level of severity. We have opted to study asthma patients classified as having mild asthma who have normal lung function and using only inhaled short-acting β-adrenergic agonists as required for relief of asthma symptoms and not using inhaled corticosteroid therapy [26]. We have avoided using patients with a more severe category of asthma because these patients are usually on chronic treatment with inhaled corticosteroids and are likely to have an inflammatory infiltrate.

We have previously demonstrated that several miRNAs are upregulated in response to inflammatory stimuli in mouse models of inflammation and in human epithelial cell lines [27], [28]. We have also shown that the lung has a very specific miRNA expression profile, which is conserved across mammalian species, and that miRNAs are important for lung development and in maintaining lung homeostasis [29], [30].

There were two main objectives of the present study. First, we compared the microRNA profile of airway biopsies from patients with mild asthma compared to those from healthy non-asthmatic volunteers. Second, we examined the effect of treatment with inhaled corticosteroid therapy over 4 weeks on the microRNA profile in patients with steroid-naïve mild asthma. We now report, for the first time, the specific miRNA expression profile in human airway biopsies from mild asthmatic patients and in the cell types commonly associated with the airways and lung. By comparing airway biopsy samples from asthmatic patients with those of healthy individuals we observed that this profile is maintained in the mild asthmatic phenotype. In addition, treatment with the corticosteroid budesonide did not affect the specific miRNA expression profile.

## Results

### MicroRNA expression was not altered in asthmatic airways

Asthma is an obstructive airway disease characterised by reversible airways contraction and associated with chronic inflammation and airway remodelling. All asthmatics had a positive skin prick test and had an FEV1 (% predicted) of 83±4. During methacholine challenge, these mild asthmatic patients had a PC_20_ of 5±1 mg/ml (table 1). The second group of patients, between the ages of 18 and 23 years, had no history of asthma. These volunteers had an FEV1 (% predicted) of 95±4 and failed to generate a response during methacholine challenge (PC_20_>16 mg/ml).

**Table 1:** Summary of patient data: healthy versus mild-asthmatic.

	n	Age (years)	Male/female ratio	FEV1 (% predicted)	PC20 (mg/ml)
Healthy	8	20±1	3/5	95±4	>16
Asthmatic	8	29±3	1/7	83±4	5±1

**Figure 1:**
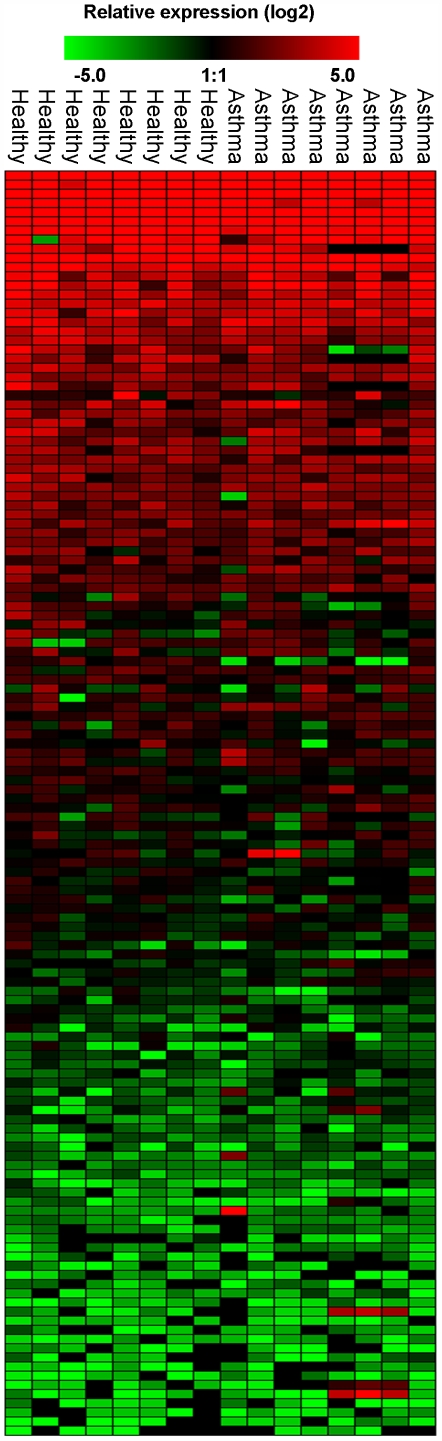
Profile of miRNA expression in control and asthmatic patients. A panel of 227 individual microRNAs were measured in airway biopsies obtained from normal and mild asthmatics patients using semi-quantitative real-time RT-PCR. The ΔCT method was used to calculate the relative expression (compared to the mean expression of all miRNAs measured) of each miRNA (2^-(ΔCT individual miRNA – mean of 227 miRNAs)^) and each sample was normalised to the snoRNA RNU44. Red indicates an increase in expression and green a decrease in expression relative to the mean expression of 227 miRNAs. Black means not detected. During the analysis, care was taken to avoid the detection of false positive, therefore ΔCT values less than 37 (that corresponded to the value of signal from the no-template control) were removed as were miRNAs that were on the limit of detectability having a sample number (n) less than n = 4. Each column represents an individual biopsy sample and each row an individual miRNA (mild asthmatic n = 8 and healthy n = 8). Statistical analysis on the sample groups was measured in Genesis using ANOVA with a p-value threshold of 0.05.

**Figure 2:**
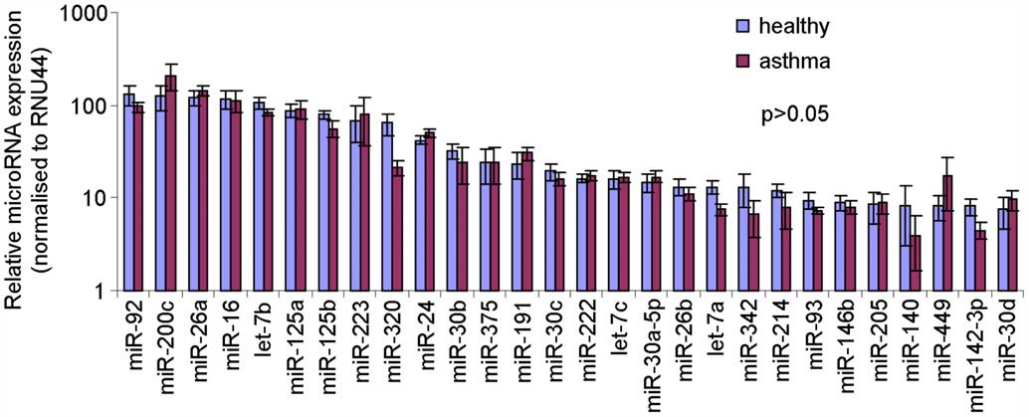
Highly expressed miRNAs did not alter in mild asthmatic airways. Individual microRNAs were measured in airway biopsies obtained from normal and mild asthmatics patients using semi-quantitative real-time RT-PCR. The ΔCT method was used to calculate the relative expression (compared to the mean expression of all miRNAs measured) of each miRNA (2^-(ΔCT individual miRNA – mean of 227 miRNAs)^) and each sample was normalised to the snoRNA RNU44. A total of 28 miRNAs had a relative expression value (2^-(ΔΔCT)^) greater than 10 and were therefore classified as being highly expressed.

Since miRNAs have been identified as key regulators of inflammatory, developmental and homeostatic gene expression, the miRNA expression profile of asthmatic airways was compared with non-asthmatic in order to identify any changes in miRNA expression. The expression of 227 miRNAs was analysed by RT-PCR from each individual biopsy. The expression of each miRNA was calibrated to the small nucleolar RNA RNU44 (house-keeping gene) and the relative expression (2^-(ΔΔCT)^) calculated using the standard ΔCT method and based on the mean expression value of all 227 miRNAs from each person (figure 1, table S1). An expression heat map of the data was produced in Genesis software (Graz University of Technology) and statistical analysis performed by ANOVA. Of the 227 miRNAs analysed no miRNAs were differentially expressed in asthmatic samples compared to healthy. This included those miRNAs that have a relatively high expression in human airways (2^-(ΔΔCT)^>10), which are likely to represent those miRNAs that are expected to have a biological function in airway cells (figure 2).

### Budesonide treatment did not alter the miRNA expression profile

Corticosteroids are commonly used to suppress the chronic inflammation underlying asthma. In the five asthmatic patients who underwent budesonide therapy for 28 days, the mean PC_20_ increased from 3.7±2.7 mg/ml to >16 mg/ml, indicating an improvement in the clinical outcome (table 2).

**Table 2:** Summary of patient data: mild-asthmatics before and after budesonide treatment.

Budesonide treatment	n	Age (years)	Male/female ratio	FEV1 (% predicted)	PC20 (mg/ml)
Before	5	29±8	1/4	89±5	3.7±2.7
After	5	29±8	1/4	85±10	>16

**Figure 3:**
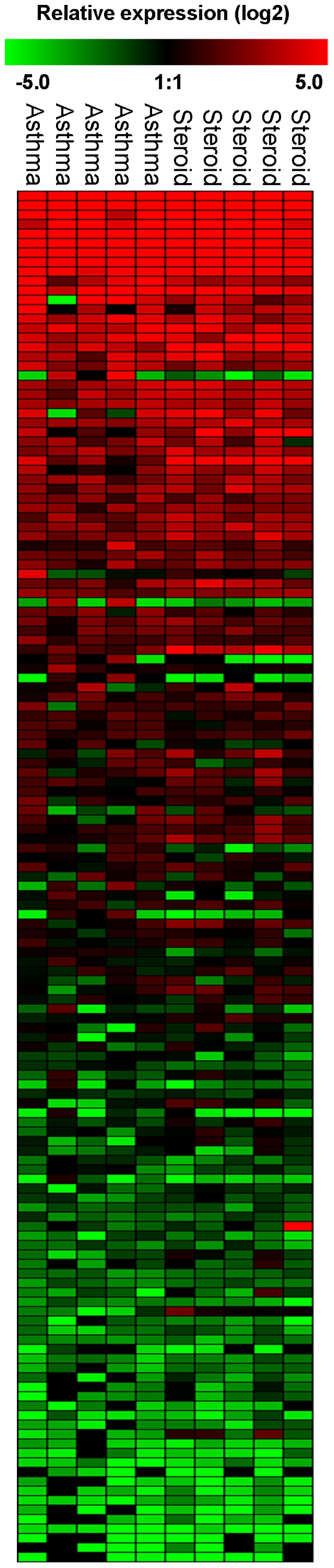
Profile of miRNA expression in asthmatic patients treated before and following treatment with budesonide. A panel of 227 individual microRNAs were measured in airway biopsies obtained from asthmatics patients before and after 28 days treatment with twice daily inhaled budesonide (200 µg) using semi-quantitative real-time RT-PCR. The ΔCT method was used to calculate the relative expression (compared to the mean expression of all miRNAs measured) of each miRNA (2^-(ΔCT individual miRNA – mean of 227 miRNAs)^) and each sample was normalised to the snoRNA RNU44. Red indicates an increase in expression and green a decrease in expression relative to the mean expression of 227 miRNAs. Black means not detected. Values with a ΔCT value of 37 or greater were removed from the analysis as were miRNAs at the limit of detection having a sample number less than n = 3. Each column represents an individual biopsy sample and each row an individual miRNA (n = 5 before budesonide and n = 5 after budesonide). Statistical analysis on the sample groups was measured in Genesis using ANOVA with a p-value threshold of 0.05.

Considering that budesonide improved the mild asthmatic phenotype, the miRNA expression profile was compared between airway biopsy samples from asthmatic patients before and after budesonide treatment. Of the 227 miRNAs measured using RT-PCR, there was no alteration in the miRNA expression profile (figure 3, table S2), with the tissue-specific miRNA expression profile persisting despite a 4-week long regime of budesonide.

### Analysis of microRNA expression profile of human airway biopsies

**Figure 4:**
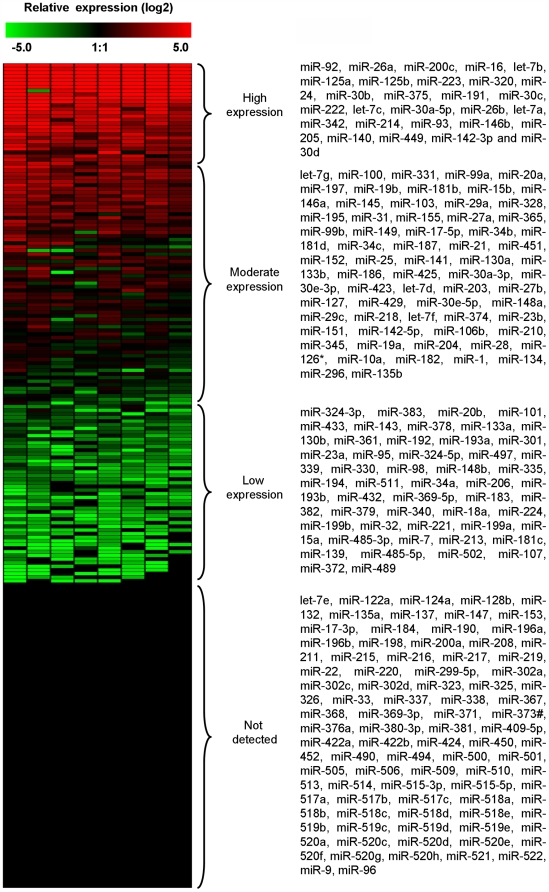
MicroRNA expression analysis of human airway biopsies. A panel of 227 individual microRNAs were measured using semi-quantitative real-time RT-PCR on non-asthmatic, healthy human airway biopsy samples (n = 8). The ΔCT method was used to calculate the relative expression (compared to the mean expression of all miRNAs measured) of each miRNA (2^-(ΔCT individual miRNA – mean of 227 miRNAs)^) and each sample was normalised to the snoRNA RNU44. Red indicates an increase in expression and green a decrease in expression relative to the mean expression of 227 miRNAs.

The interaction between miRNAs and mRNA are important in regulating gene expression and maintaining tissue homeostasis. It is often the case that a diseased phenotype is associated with an altered miRNA expression profile. Individual tissues and cell types can also be identified based on their particular miRNA expression profile and express tissue specific miRNAs. Therefore, the miRNA expression profile from non-asthmatic, healthy human airways was analysed in order to identify airway specific miRNAs (n = 8). The expression of 227 miRNAs were ranked from the most highly expressed to the least expressed and were then grouped into four separate categories based on their expression levels; high expression (2^-(ΔΔCT)^>10), moderate expression (2^-(ΔΔCT)^ = 0.5–10), low expression (2^-(ΔΔCT)^<0.5) and no expression (not detected) (figure 4). This analysis revealed that miR-92, miR-26a, miR-200c, miR-16, let-7b, miR-125a, and miR-125b were the most highly expressed in human airway tissue, having levels more than 70-fold higher than the average miRNA and contributing 55.5% of the total mRNA detected in airway biopsies. In addition, members of the let-7 family (let-7a, let-7b and let-7c), miR-26 family (miR-26a and miR-26b), miR-125 family (miR-125a and miR-125b) and miR-30 family (miR-30a-5p, miR-30b and miR-30c) also fell within the highly expressed category and are therefore considered to be human airway specific (figure 4).

**Figure 5:**
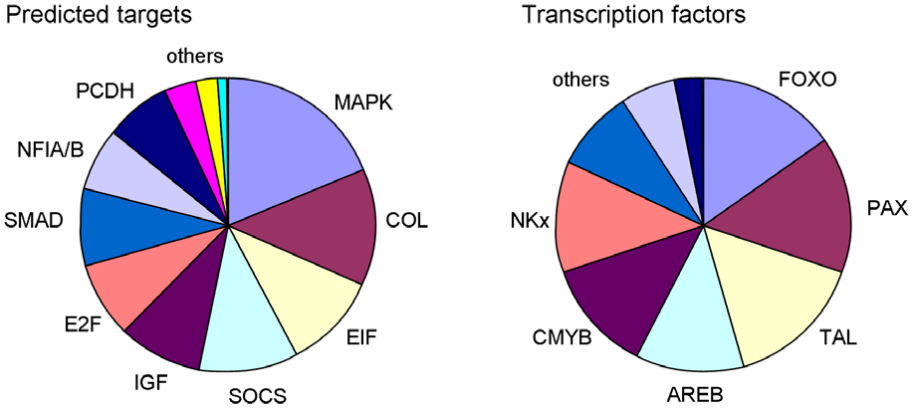
Bioinformatic analysis of highly expressed miRNAs in human airways. The specific miRNA targets were predicted using Targetscan 4.2. The gene targets for those miRNAs highly expressed in the airway (2^-(ΔΔCT)^>10). Targets that shared a high frequency across the miRNAs analysed (28 miRNAs in the highly expressed group) are plotted. Likewise, the transcription factor binding sites within 5 kb of the miRNA start site, for the same set of miRNAs, were analysed using UCSC Genome Browser. Abbreviations; MAPK, mitogen activated protein kinase; COL, collagen; EIF, eukaryotic translation initiation factor; SOCS, suppressor of cytokine signalling; IGF, insulin-like growth factor; PCDH, protocadherin.

Target prediction and validation for miRNAs is often problematic due to the number of potential mRNA targets that current prediction algorithms identify. In order to relate the specific miRNA expression profile in human airways to potential mRNAs targets, we analysed all highly expressed miRNAs using an available public database, Targetscan 4.2 (http://www.targetscan.org). For each highly expressed miRNA the chromosome location, number of potential targets and example targets (based on those targets that may be involved in signalling pathways, transcription factors or genes related to airway structure/function) have been analysed (table S3). In addition, the transcription factor binding sites that occur within 5 kb upstream of the miRNA gene start site have been analysed using the UCSC Genome Browser tool (http://genome.ucsc.edu). Those miRNAs that were highly expressed were not associated with a particular chromosomal location; rather they are located at various sites throughout the genome (table S3). The highly expressed miRNAs most frequently targeted MAP kinases such as *MAPK6* and *MAP4K4* and also pro-collagen transcripts such as *COL1A2*, transcripts associated with the translational machinery such as *E2F3*, *E2F7* and *EIF4G2* and transcription factors such as *NFIA*, *NFIB*. The transcription factor binding sites (up to 5 kb upstream of the miRNA start sites) included sites for FOXO, PAX5, TAL1 and AREB transcription factors (figure 5).

### Cell-specific microRNA expression

In order to determine the expression of miRNAs in isolated cell types, qRT-PCR was performed on various cell types from the airways and lungs. The cell types analysed included alveolar epithelial cells, bronchial epithelial cells, airway smooth muscle cells, alveolar macrophages and bronchial fibroblasts. The expression of several miRNAs was also analysed from whole human lung samples, thus representing a range of cell types that have critical functions within the airways and lungs. The miRNAs that were measured were those that had a level of expression 10-fold or higher than the average expression within human airways (figure 2).

**Figure 6:**
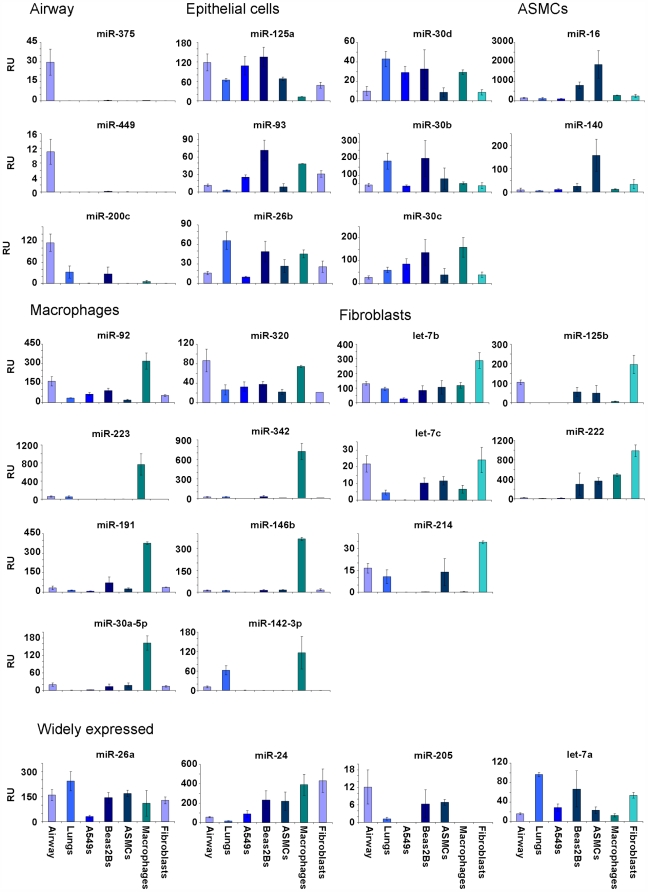
Differential expression of highly expressed miRNAs in individual airway and lung cell populations. The expression of miRNAs that had an expression value 10-fold or more above the average miRNA expressed in the airway, were analysed in airway biopsies, whole lung samples, alveolar epithelial cells (A549), bronchial epithelial cells (Beas2B), airway smooth muscle (ASM), alveolar macrophages (Macs) and bronchial fibroblasts (Fibs). The relative expression of each miRNA was calculated using the ΔΔCT method (calibrated to the mean value of each miRNA in the airway) generating a fold-difference in expression compared to the airways (relative expression, RU). The expression of each miRNA was attributed a grouping according to each cell population (epithelial cells (A549s or Beas2Bs), airway smooth muscle cells (ASMCs), alveolar macrophages or bronchial fibroblasts) or to whole airways or to a group having a wide distribution.

The relative expression (RU) for each miRNA was calculated using the ΔΔCT method and the airway samples used as the calibrator to determine fold-differences in expression (figure 6). Three miRNAs, miR-375, miR-449 and miR-200c were specifically expressed only in the airway samples. The expression of miRNAs in epithelial cells was similar to that in whole lung and included miR-125a, miR-30b/c/d, miR-20d, miR-93 and miR-26b, although some of the miRNAs were also significantly expressed in macrophages and fibroblasts (e.g. miR-30c). Two miRNAs were highly expressed in airway smooth muscle cells, miR-16 and miR-140. Alveolar macrophages possessed the highest number of highly expressed miRNAs than the other cell types and included miR-92, miR-223, miR-191, miR-30a-5p, miR-320, miR-342, miR-146b and miR-142-3p. Those miRNAs that were dominant in lung fibroblasts were let-7b, let-7c, miR-214, miR-125b and miR-222, while the remaining miRNAs demonstrated a more general expression pattern across most cell types (figure 6).

## Discussion

MicroRNAs are a recently discovered family of non-coding RNA molecules that post-transcriptionally regulate gene expression. They are endogenously expressed as primary-miRNAs and are processed in the nucleus and cytoplasm to form ∼22 nt double-stranded miRNAs. Only one strand of the miRNA is incorporated into a ribonucleoprotein complex called the miRISC, where it binds to its target mRNA and either prevents protein translation or initiates mRNA destabilisation. The regulatory potential of miRNAs is vast when you consider that most miRNAs are predicted to target many 100s of mRNAs [31]. Although ∼700 miRNAs have been identified in the human genome, the biological function of the majority of these is unknown. Furthermore, it is unclear as to how miRNAs contribute to complex diseases such as asthma. To measure the miRNA expression profile, we used semi-quantitative RT-PCR method based on highly sensitive TaqMan technology (Applied Biosystems). This allows for small quantities of total RNA to be used for the measurement of many individual miRNA molecules. This technique is therefore appropriate for the analysis of small airway biopsies, which contain small amounts of starting RNA. Of the 227 miRNAs that were measured no differences in expression levels were observed between healthy and mild asthmatic samples. Despite the lack of differential expression between healthy and asthmatic biopsies, this study has revealed a number of important issues regarding such analysis. The main problem/issue with analysing the miRNA expression in airway biopsies is the degree of cellular heterogeneity within each biopsy [32]–[34]. This has the potential of masking any differences in the expression of individual miRNAs within specific cell types. Therefore, there remains the possibility that differential miRNA expression is occurring in the airways of mild-asthmatics but within a particular cell type. In addition, a significant change might be observed by using biopsies from more severe asthma patients, although interpretation maybe confounded by the presence of inflammatory cells such as eosinophils, mast cells and lymphocytes. Indeed non-resident, migratory cells that enter sites of inflammation in the airway would augment the heterogeneity of biopsy samples.

The analysis of individual cell types from the airways and lungs of humans did indeed reveal that the expression profile of each miRNA differed significantly depending on the cell type. This differential expression is an important consideration when analysing samples such as airway biopsies that have a heterogeneous cell population. These findings gave an indication as to which cell types contributed the most to the overall expression level of an individual miRNA within the airways, even though all 26 miRNAs were expressed 10-fold or more higher than average. For example, miR-140 was predominantly expressed in airway smooth muscle cells. The most prominent cell type was alveolar macrophages, which possessed a high expression of several miRNAs including miR-223 and miR-146b, further emphasising how migratory cells (as well as resident cells) contribute to the overall miRNA expression profile within a given tissue. These two miRNAs have previously been shown to be specifically expressed in myeloid and lymphoid cell lineages [35] and may function as regulators of the innate immune system [27]. Two miRNAs, miR-375 and miR-449 were specific only to airway samples, indicating that these miRNAs are expressed in a cell type that is explicit the airways.

The use of drugs such as β_2_ agonists and corticosteroids by these patients may also interfere with the expression of miRNAs that may be involved in the pathophysiology of asthma; however, we examined asthmatics who have never received corticosteroid therapy for their asthma. The authors also appreciate that despite the identification of >700 human miRNAs, we were only able to analysis the expression of 227 miRNAs. This was a result of the small quantities of RNA that can be obtained from biopsies, which required the use the RT-PCR based approach. However, the miRNAs included in the panel represent most of those that were recently shown to be either ubiquitously expressed or those that account for most of the differences in miRNA profiles between cell lineages and tissues [36]. Indeed, this conclusion is demonstrated in the supplemental Tables where we failed to observe the expression of the most recently identified miRNAs i.e. miR-500 upwards.

To investigate the effect of inhaled corticosteroids, similar miRNA expression analysis was performed on asthmatic airway biopsy samples following budesonide treatment and compared to pre-treatment samples. We ensured that the patients had administered the correct dose of inhaled medication by counting the appropriate number of puffs on the inhaler counter. As a positive indication that budesonide was correctly administered, there was an improvement in bronchial hyperresponsiveness into the normal range, as would be expected with this treatment in patients with mild asthma [37]. We deliberately chose to study patients who have not been on treatment with inhaled corticosteroids in order to avoid the potential issue of tachyphylaxis to this treatment. Corticosteroids are potent anti-inflammatory agents that are known to interfere with a number of intracellular pathways including transcription factors such as NF-κB and AP-1 and to inhibit many pro-inflammatory cytokines and chemokines [38], [39]. These anti-inflammatory effects of corticosteroids are likely to underlie the improvement in bronchial hyperresponsiveness observed. Despite some of the highly expressed miRNAs containing NF-κB transcription factor binding sites, no alteration in miRNA expression was observed following budesonide treatment. Considering the lack of differential expression of miRNAs in mild asthmatics, it may be that budesonide does not exert an action upon basal miRNA expression. Again, perhaps a severe asthmatic phenotype would reveal the effects of budesonide. However, our previous studies examining the miRNA expression in lung tissue from mice challenged with LPS revealed no detectable effects following treatment with dexamethasone [40].

Expression profiling of human airway biopsies revealed several highly expressed miRNAs, including miR-92, miR-200c, miR-26a, miR-16, let-7b, miR-125a and miR-125b, which together comprised 55.5% of the total miRNA species analysed. These miRNAs are likely to represent those that have a significant influence on gene expression in the airways. For instance, miR-26a has been shown to target the transcription factor SMAD1 during the differentiation of stem cells [41] and we have previous shown that this is selectively expressed in the bronchial and alveolar epithelial cells in mouse lung [28]. SMAD1 is known to be an important transcription factor for the regulation of bone morphogenic protein signalling during lung development and pulmonary vascular remodelling [42], [43]. This suggests that miR-26a might be important in controlling essential developmental and physiological events in the lung, which may also include pathological events during pulmonary diseases such as asthma. Indeed, we have previously demonstrated that it is upregulated in the adult lung following the postnatal period of lung development and is highly expressed within bronchial and alveolar epithelial cells [29]. The rate of apoptosis or proliferation is known to be regulated by miR-16 and let-7 [23], [44] whilst miR-125 is the mammalian homologue of lin-4, and has previously been shown to be associated with embryonic development [45]. The development and maintenance of airway tissue and the regulation of gene expression in the airway is therefore likely to be under the control of these miRNAs.

Bioinformatic analysis of the transcription factor binding sites in the promoter regions located upstream of miRNA start sites, provides information on which transcription factors are involved in the regulation of miRNA expression. However, no transcription factor binding sites were particularly overrepresented in the 5′-regions upstream of the highly expressed miRNA start sites. Furthermore, the predicted targets of these highly expressed miRNA may to some extent reveal their biological function. For example, several MAP kinases were predicted to be targets, suggesting that these highly expressed miRNAs may be able to inhibit signalling molecules involved in the inflammatory response.

In conclusion, we showed that changes in miRNA expression are not associated with the mild asthma phenotype. It is possible that the inflammatory changes were too mild, and that studying more severe asthma patients may reveal changes in miRNA expression. However, these patients are invariably treated with corticosteroids that may alter miRNA expression. Another approach would be to study the airways after an allergen challenge that is known to intensify allergic airway inflammation. We also found that the anti-inflammatory effects of the corticosteroid budesonide does not appear to be mediated through changes in miRNA expression, which may also be due to the mild state of inflammation in these mild asthma patients.

## Materials and Methods

### Patient assessment

Eight patients with asthma and eight normal volunteers were recruited for the study. All patients gave a history of intermittent wheeze and were only using salbutamol inhaler on an intermittent basis, less than twice a week, were corticosteroid-naïve, and all had positive skin-prick tests to common aeroallergens. These mild asthmatic patients had a PC_20_ of 5±1 mg/ml. The normal volunteers have never had any history of wheezing or chest tightness on questioning and all had PC_20_ to methacholine of greater than 16 mg/ml, and 3 of the 8 normal subjects had positive skin prick tests. The asthmatics had an FEV_1_ of 83±4% of predicted values and the normal volunteers all had FEV_1_ values 95±4% of predicted values. All normal subjects had PC_20_ methacholine greater than 16 mg/ml. None of the subjects were or had been cigarette smokers.

Each subject underwent a baseline fiberoptic bronchoscopic study using topical anesthesia with lidocaine to the upper and lower airways under sedation with intravenous midazolam (3–6 mg) and alfentanyl (125 µg). Airway biopsies were taken from segmental and sub-segmental airways of the right lower lobe and were then snap-frozen in pre-cooled isopentane in liquid nitrogen and stored at −70°C. Five of the 8 patients with asthma undertook to inhale budesonide dry powder from a Pulmicort Turbohaler^R^ (200 µg per inhalation; 2 inhalations) twice daily for 4 weeks. Compliance to treatment was ensured by (i) a phone call to the subject once a week and by (ii) asking the subject to bring back their Turbohaler inhaler and making sure that the counter shows that an appropriate number of puffs had been dispensed. At the end of 28 days, PC_20_ to methacholine was measured, fibre-optic bronchoscopy was repeated and airway biopsies were obtained as previously described. None of the patients reported any adverse events during the treatment or the bronchoscopy procedure. Ethical approval was given by the ethical review committee at Imperial College London, Royal Brompton Campus and written consent has been received from all patients involved in the study.

### qRT-PCR

The miRNA expression profile for each airway biopsy sampled was analysed using Applied Biosystems miRNA TaqMan panel of 227 individual miRNAs. The reverse transcription used 2 ng of starting total RNA for each miRNA assay. Multiplex pools of up to 48 miRNAs were used for the RT reactions according to the manufacturers' guidelines (Applied Biosystems). This enables up to 48 miRNAs to be prepared in a single RT reaction. The PCR reaction comprised 5 µl TaqMan 2× Universal PCR Master Mix, No AmpErase UNG (Applied Biosystems), 3.835 µl RNAse free water (Promega), 0.5 µl TaqMan probe (Appled Biosystems) plus 0.67 µl RT product and the reaction conditions were 95°C for 10 min and then 40 cycles of 95°C for 10 seconds and 60°C for 1 min. The amount of RNA from each sample was calibrated to the expression of the small nucleolar RNA RNU44 (house-keeping gene). This then gave a delta CT (ΔCT) value for each miRNA (miRNA CT value – RNU44 CT value). The relative expression of each miRNA was calculated by comparing each value to the mean value of all 227 miRNAs. The fold difference in expression was calculated as 2^-(ΔCT sample-ΔCT mean sample)^. In order to avoid the possibility of generating false positive values any ΔCT value of 37 or greater was removed from the data set. This value corresponded to the sample detection from the no-template control, a value which falls below the acceptable value of detection. Furthermore, any miRNA that was on the limit of detection and having a sample number less than n = 4 (or n = 3 from the budesonide study) was also removed from further analysis. Any miRNA that was not detected was regarded as not being significantly expressed in human airways. The generation of heat maps for miRNA expression and calculation of statistical significance was performed using Genesis software (Graz University of Technology) with a p-value threshold of 0.05. Relative expression of miRNAs from airway biopsies taken from asthmatics before and after budesonide treatment was also calculated using the ΔCT method (normalised to RNU44). False positives were eliminated as described and the relative expression calculated using the ΔCT method.

### Bioinformatic analysis of microRNAs

The precise genomic location and nucleotide sequence of each human miRNA was determined using miRBase (Wellcome Trust Sanger Institute; http://microrna.sanger.ac.uk). Analysis of miRNA targets was performed using the public database, Targetscan 4.2 (http://www.targetscan.org). Particular interest was given to transcription factors, signalling molecules and structural proteins that may be involved in the airways or lungs. Targets having a total context score of −0.30 or less were ignored. Evolutionarily conserved transcription factor binding sites within 5 kb of the miRNA start site were determined using University of California Santa Cruz Genome Browser (http://genome.ucsc.edu/).

### Isolation and culture of human airway and lung cells

Human adult lung was obtained from AMS Biotechnology (5 donor pool from 23, 23, 26, 29 and 36 years old), and the RNA was extracted using a modified guanidine thiocyanate technique (Chomczynski & Sacchi, 1987). The alveolar A549 epithelial cell line was grown in Dulbecco's Modified Eagles Medium containing 10% fetal calf serum (FCS) and 5% L-glutamine in a humidified chamber in 5% CO_2_. Prior to RNA isolation cells were washed twice in Hanks Balanced Salt Solution and removed from the flask using 10% Trypsin diluted in Hanks Balanced Salt Solution incubated at 37°C. Cells were centrifuged at 1200 RCF to obtain a cell pellet and then resuspended in lysis buffer (miRVana, Ambion). RNA extraction was performed as described above according to the manufacturer's guidelines. The bronchial Beas2B epithelial cell line was grown in Keratinocyte serum free medium (Invitrogen) containing 5% L-glutamine, epidermal growth factor and bovine pituitary extract (Invitrogen). Cells were recovered and the RNA extracted as described.

Airway smooth muscle cells (ASMCs) were obtained from human lungs (resected). Lungs were dissected to reveal the muscle bundles, which were then remove by plucking with forceps and placed in antibiotic-containing (penicillin and streptomycin) Dulbecco's Modified Eagles Medium containing 5 ml sodium pyruvate, 5 ml MEM non essential amino acids 100×, L-glutamine and 10% FCS (all Sigma). Explants were centrifuged at 1200 RCF and resuspended in 25 ml of medium and incubated at 37°C in a humidified chamber. The media was changes after 10 days and the cells grown until 75% confluent. Cells were removed by scraping and the RNA extracted as described above. Human alveolar macrophages were isolated using a sterile saline solution (0.9%) (Baxter Healthcare) that was perfused through resected lung airways from patients with lung carcinoma using a syringe and cannula. The washout was centrifuged at 400 g for 10 min to collect cells and re-suspended in RPMI media, before being layered on top of a Ficoll-Paque column (Sigma-Aldrich). Lymphocytes were harvested from the Ficoll/plasma interface after the column had been centrifuged at 400 g for 30 min at room temperature. The lymphocytes were washed in fresh media before being left to adhere for 2 h in a 37°C incubator. After this time non-adherent cells were removed and the adherent macrophages washed with media. Cells were recovered and the RNA was extracted as described above. Fibroblasts were extracted from bronchial biopsies, which were collected from healthy non-smoking volunteers undergoing bronchoscopy at Wythenshawe Hospital, Manchester following full ethical approval and consent. Primary human bronchial fibroblasts were isolated from bronchial biopsies by explant culture in supplemented DMEM with 10%FCS.

### Statistics

The fold-change in microRNA expression between asthmatic and non-asthmatic airway biopsies was measured by ANOVA using Genesis software provide by the Graz University of Technology (Switzerland), using a p-value threshold of 0.05. Statistical significance was validated using a two-tailed Student's t-test assuming unequal variance, whereby significance was achieved for p<0.05.

## Supporting Information

Table S1 Relative expression values for healthy and asthma biopsy samples.
(0.42 MB DOC)

Table S2 Relative expression values for asthma biopsy samples before and after budesonide treatment.
(0.42 MB DOC)

Table S3 Bioinformatic analysis of highly expressed miRNAs expressed in airway biopsies.
(0.05 MB DOC)
