# Supplementary material for: MicroRNA Expression Profiling in Mild Asthmatic Human Airways and Effect of Corticosteroid Therapy
Source: PLoS One. 2009 Jun 12;4(6):e5889. doi: 10.1371/journal.pone.0005889 (PMC2690402; doi:10.1371/journal.pone.0005889)
Supplement: Table S1 — Relative expression values for healthy and asthma biopsy samples. (0.42 MB DOC) [file pone.0005889.s001.doc]

**Table S1.**

| *Healthy* |  |  |  | *Mild-asthmatic* |  |  |  |
| --- | --- | --- | --- | --- | --- | --- | --- |
| *miRNA* | *2^-(Δ∆CT^)* | *SEM* | *sample n* | *miRNA* | *2^-(Δ∆CT^)* | *SEM* | *sample n* |
| let-7a | 12.43598 | 2.200924 | 8 | let-7a | 7.443544 | 1.109311 | 8 |
| let-7b | 94.50935 | 15.86432 | 8 | let-7b | 84.50535 | 8.153965 | 8 |
| let-7c | 15.24388 | 3.521647 | 8 | let-7c | 16.89917 | 2.276269 | 8 |
| let-7d | 2.377245 | 0.370685 | 8 | let-7d | 0.880222 | 0.05971 | 8 |
| let-7e | ND |  |  | let-7e | ND |  |  |
| let-7f | 2.029442 | 0.217365 | 8 | let-7f | 1.236535 | 0.265794 | 8 |
| let-7g | 7.674613 | 2.020972 | 8 | let-7g | 4.520224 | 0.676844 | 8 |
| miR-1 | 1.420822 | 0.176131 | 8 | miR-1 | 0.290089 | 0.09382 | 8 |
| miR-100 | 7.282339 | 1.289543 | 8 | miR-100 | 8.38015 | 1.750036 | 8 |
| miR-101 | 1.256449 | 0.098563 | 8 | miR-101 | 0.443701 | 0.078228 | 7 |
| miR-103 | 4.874356 | 0.667596 | 8 | miR-103 | 3.753434 | 0.643662 | 8 |
| miR-106b | 1.807853 | 0.123503 | 8 | miR-106b | 1.093982 | 0.254085 | 8 |
| miR-107 | 0.851083 | 0.004693 | 5 | miR-107 | 0.082511 | 0.022323 | 7 |
| miR-10a | 1.528788 | 0.126249 | 8 | miR-10a | 0.994442 | 0.295752 | 8 |
| miR-122a | ND |  |  | miR-122a | ND |  |  |
| miR-124a | ND |  |  | miR-124a | ND |  |  |
| miR-125a | 79.94703 | 15.51254 | 8 | miR-125a | 91.53247 | 19.93111 | 8 |
| miR-125b | 71.32124 | 7.216998 | 8 | miR-125b | 55.86367 | 10.86896 | 8 |
| miR-126* | 1.558526 | 0.110936 | 8 | miR-126* | 1.323115 | 0.30353 | 8 |
| miR-127 | 2.169291 | 0.287054 | 8 | miR-127 | 2.208043 | 0.702918 | 7 |
| miR-128b | ND |  |  | miR-128b | ND |  |  |
| miR-130a | 2.681298 | 0.208918 | 8 | miR-130a | 2.278423 | 0.354515 | 8 |
| miR-130b | 1.166657 | 0.057337 | 8 | miR-130b | 0.462972 | 0.076617 | 8 |
| miR-132 | ND |  |  | miR-132 | ND |  |  |
| miR-133a | 1.168873 | 0.095463 | 8 | miR-133a | 1.303834 | 0.579383 | 7 |
| miR-133b | 2.643246 | 0.493586 | 8 | miR-133b | 1.596573 | 0.340693 | 7 |
| miR-134 | 1.39899 | 0.126269 | 8 | miR-134 | 0.464629 | 0.239558 | 5 |
| miR-135a | ND |  |  | miR-135a | ND |  |  |
| miR-135b | 1.342282 | 0.143344 | 8 | miR-135b | 0.570042 | 0.174258 | 8 |
| miR-137 | ND |  |  | miR-137 | ND |  |  |
| miR-139 | ND |  |  | miR-139 | ND |  |  |
| miR-140 | 8.213148 | 5.182217 | 8 | miR-140 | 3.984376 | 2.331959 | 8 |
| miR-141 | 2.704134 | 0.677258 | 8 | miR-141 | 3.762239 | 0.877351 | 8 |
| miR-142-3p | 8.085028 | 1.702325 | 8 | miR-142-3p | 4.519057 | 0.866187 | 8 |
| miR-142-5p | 1.880291 | 0.229738 | 8 | miR-142-5p | 0.592961 | 0.246747 | 7 |
| miR-143 | 1.215016 | 0.096166 | 8 | miR-143 | 0.491773 | 0.154081 | 6 |
| miR-145 | 5.676738 | 1.186869 | 8 | miR-145 | 12.95787 | 3.574784 | 8 |
| miR-146a | 5.836638 | 0.777806 | 8 | miR-146a | 4.536205 | 0.751028 | 8 |
| miR-146b | 8.942118 | 1.725147 | 8 | miR-146b | 7.963313 | 1.36302 | 8 |
| miR-147 | ND |  |  | miR-147 | ND |  |  |
| miR-148a | 2.112391 | 0.606818 | 8 | miR-148a | 1.316052 | 0.337638 | 8 |
| miR-148b | 1.010848 | 0.011303 | 8 | miR-148b | 124.4323 | 124.2192 | 7 |
| miR-149 | 3.382606 | 1.360989 | 8 | miR-149 | 1.76168 | 0.306115 | 8 |
| miR-151 | 1.881099 | 0.150897 | 8 | miR-151 | 1.538848 | 0.401898 | 5 |
| miR-152 | 2.921288 | 0.337931 | 8 | miR-152 | 1.901988 | 0.220474 | 8 |
| miR-153 | ND |  |  | miR-153 | ND |  |  |
| miR-155 | 4.175984 | 0.853258 | 8 | miR-155 | 3.394973 | 0.650867 | 7 |
| miR-15a | 0.916491 | 0.010645 | 7 | miR-15a | 0.098636 | 0.030091 | 7 |
| miR-15b | 5.934993 | 1.268964 | 8 | miR-15b | 5.412294 | 1.208881 | 8 |
| miR-16 | 104.2011 | 25.9515 | 8 | miR-16 | 112.4525 | 30.27876 | 8 |
| miR-17-3p | ND |  |  | miR-17-3p | ND |  |  |
| miR-17-5p | 3.270119 | 1.171826 | 8 | miR-17-5p | 1.858615 | 0.657745 | 8 |
| miR-181b | 5.94786 | 1.04652 | 8 | miR-181b | 5.186914 | 0.834778 | 8 |
| miR-181c | 0.896534 | 0.029739 | 5 | miR-181c | 0.058282 | 0.028923 | 6 |
| miR-181d | 3.171362 | 0.842682 | 8 | miR-181d | 2.521351 | 0.508753 | 7 |
| miR-182 | 1.527712 | 0.103819 | 8 | miR-182 | 1.006688 | 0.161017 | 8 |
| miR-183 | 0.960627 | 0.037674 | 5 | miR-183 | 0.119729 | 0.038708 | 7 |
| miR-184 | ND |  |  | miR-184 | ND |  |  |
| miR-186 | 2.598923 | 0.388358 | 8 | miR-186 | 1.906443 | 0.450916 | 8 |
| miR-187 | 3.013507 | 0.47115 | 7 | miR-187 | 0.440609 | 0.243917 | 7 |
| miR-18a | 0.940806 | 0.006772 | 7 | miR-18a | 0.184855 | 0.136039 | 6 |
| miR-190 | ND |  |  | miR-190 | ND |  |  |
| miR-191 | 21.63938 | 7.597605 | 8 | miR-191 | 30.41565 | 5.172285 | 8 |
| miR-192 | 1.089134 | 0.072261 | 7 | miR-192 | 0.300632 | 0.057933 | 8 |
| miR-193a | 1.074611 | 0.041046 | 7 | miR-193a | 0.216446 | 0.06241 | 7 |
| miR-193b | 0.973623 | 0.03717 | 7 | miR-193b | 0.124684 | 0.041355 | 4 |
| miR-194 | 1.00473 | 0.029319 | 7 | miR-194 | 0.207594 | 0.040534 | 7 |
| miR-195 | 4.543958 | 1.213431 | 8 | miR-195 | 4.324881 | 0.978373 | 8 |
| miR-196a | ND |  |  | miR-196a | ND |  |  |
| miR-196b | ND |  |  | miR-196b | ND |  |  |
| miR-197 | 6.681176 | 2.213281 | 8 | miR-197 | 6.410303 | 0.897381 | 8 |
| miR-198 | ND |  |  | miR-198 | ND |  |  |
| miR-199a | 0.917347 | 0.01274 | 7 | miR-199a | 0.110412 | 0.025428 | 5 |
| miR-199b | 0.934212 | 0.014323 | 7 | miR-199b | 0.097112 | 0.02153 | 7 |
| miR-19a | 1.736255 | 0.203096 | 8 | miR-19a | 0.857864 | 0.184051 | 8 |
| miR-19b | 6.606011 | 0.902584 | 8 | miR-19b | 7.307341 | 0.984313 | 8 |
| miR-200a | ND |  |  | miR-200a | ND |  |  |
| miR-200c | 111.1977 | 36.29668 | 8 | miR-200c | 211.5118 | 66.40578 | 8 |
| miR-203 | 2.203486 | 0.2051 | 8 | miR-203 | 2.026336 | 0.988664 | 8 |
| miR-204 | 1.707161 | 0.356365 | 8 | miR-204 | 0.721844 | 0.159348 | 8 |
| miR-205 | 8.398609 | 3.260017 | 8 | miR-205 | 8.896547 | 2.189153 | 5 |
| miR-206 | 0.978856 | 0.047575 | 7 | miR-206 | 0.181137 | 0.088532 | 4 |
| miR-208 | ND |  |  | miR-208 | ND |  |  |
| miR-20a | 6.897449 | 1.103873 | 8 | miR-20a | 5.682053 | 0.621712 | 8 |
| miR-20b | 1.285792 | 0.203916 | 8 | miR-20b | 0.225368 | 0.048226 | 7 |
| miR-21 | 3.007527 | 0.517341 | 8 | miR-21 | 2.849014 | 0.484273 | 8 |
| miR-210 | 1.771906 | 0.185597 | 8 | miR-210 | 1.03286 | 0.241349 | 8 |
| miR-211 | ND |  |  | miR-211 | ND |  |  |
| miR-213 | 0.914785 | 0.026394 | 6 | miR-213 | 7.404994 | 4.174206 | 8 |
| miR-214 | 11.69667 | 2.038667 | 8 | miR-214 | 8.012058 | 3.334355 | 5 |
| miR-215 | ND |  |  | miR-215 | ND |  |  |
| miR-216 | ND |  |  | miR-216 | ND |  |  |
| miR-217 | ND |  |  | miR-217 | ND |  |  |
| miR-218 | 2.032741 | 0.466861 | 6 | miR-218 | 9.279136 | 5.589006 | 7 |
| miR-219 | ND |  |  | miR-219 | ND |  |  |
| miR-22 | ND |  |  | miR-22 | ND |  |  |
| miR-220 | ND |  |  | miR-220 | ND |  |  |
| miR-220 | ND |  |  | miR-220 | ND |  |  |
| miR-221 | 0.923091 | 0.049166 | 5 | miR-221 | 0.182482 | 0.065567 | 7 |
| miR-222 | 15.35223 | 1.645664 | 8 | miR-222 | 17.39764 | 2.002936 | 8 |
| miR-223 | 62.56191 | 30.41448 | 8 | miR-223 | 79.36734 | 42.37868 | 8 |
| miR-224 | 0.934865 | 0.065987 | 5 | miR-224 | 0.121207 | 0.025855 | 7 |
| miR-23a | 1.057889 | 0.067167 | 7 | miR-23a | 0.41327 | 0.135029 | 8 |
| miR-23b | 1.940234 | 0.235298 | 8 | miR-23b | 0.904217 | 0.185809 | 8 |
| miR-24 | 38.36487 | 3.791606 | 8 | miR-24 | 50.21102 | 5.95996 | 8 |
| miR-25 | 2.807686 | 0.464315 | 8 | miR-25 | 2.208944 | 0.548938 | 8 |
| miR-26a | 110.2189 | 22.5677 | 8 | miR-26a | 146.0427 | 18.1599 | 8 |
| miR-26b | 12.64987 | 2.842028 | 8 | miR-26b | 11.04166 | 1.743578 | 8 |
| miR-27a | 3.825044 | 0.293816 | 8 | miR-27a | 5.245191 | 0.914432 | 8 |
| miR-27b | 2.200781 | 0.257441 | 8 | miR-27b | 1.570247 | 0.289175 | 8 |
| miR-28 | 1.618758 | 0.170973 | 8 | miR-28 | 0.354805 | 0.140487 | 7 |
| miR-296 | 1.381657 | 0.108865 | 8 | miR-296 | 0.431694 | 0.114155 | 8 |
| miR-299-5p | ND |  |  | miR-299-5p | ND |  |  |
| miR-29a | 4.753755 | 0.609711 | 8 | miR-29a | 5.133481 | 1.051363 | 8 |
| miR-29c | 2.069036 | 0.183173 | 8 | miR-29c | 1.526044 | 0.485135 | 8 |
| miR-301 | 1.071035 | 0.049051 | 8 | miR-301 | 0.245485 | 0.04272 | 8 |
| miR-302a | ND |  |  | miR-302a | ND |  |  |
| miR-302c | ND |  |  | miR-302c | ND |  |  |
| miR-302d | ND |  |  | miR-302d | ND |  |  |
| miR-30a-3p | 2.511203 | 0.353616 | 8 | miR-30a-3p | 3.613345 | 1.320095 | 8 |
| miR-30a-5p | 13.92956 | 3.351006 | 8 | miR-30a-5p | 16.91744 | 2.405969 | 8 |
| miR-30b | 29.56895 | 5.500053 | 8 | miR-30b | 224.8857 | 196.9362 | 8 |
| miR-30c | 18.20466 | 3.840048 | 8 | miR-30c | 16.09955 | 2.645567 | 8 |
| miR-30d | 7.520817 | 2.788018 | 8 | miR-30d | 9.709207 | 2.365101 | 8 |
| miR-30e-3p | 2.508876 | 0.459282 | 8 | miR-30e-3p | 3.415753 | 0.924538 | 8 |
| miR-30e-5p | 2.151573 | 0.193253 | 8 | miR-30e-5p | 1.304688 | 0.311589 | 8 |
| miR-31 | 4.434842 | 1.260032 | 8 | miR-31 | 4.637581 | 1.212934 | 8 |
| miR-32 | 0.930589 | 0.022398 | 6 | miR-32 | 0.039899 | 0.011886 | 5 |
| miR-320 | 57.79706 | 17.42947 | 8 | miR-320 | 21.01124 | 3.815878 | 5 |
| miR-323 | ND |  |  | miR-323 | ND |  |  |
| miR-324-3p | 1.290187 | 0.167564 | 8 | miR-324-3p | 0.504937 | 0.1562 | 8 |
| miR-324-5p | 1.044 | 0.018689 | 7 | miR-324-5p | 0.220862 | 0.050948 | 8 |
| miR-325 | ND |  |  | miR-325 | ND |  |  |
| miR-326 | ND |  |  | miR-326 | ND |  |  |
| miR-328 | 4.686899 | 1.216818 | 8 | miR-328 | 4.706919 | 1.095139 | 8 |
| miR-33 | ND |  |  | miR-33 | ND |  |  |
| miR-330 | 1.02652 | 0.060887 | 6 | miR-330 | 0.125459 | 0.051837 | 8 |
| miR-331 | 7.255398 | 1.271796 | 8 | miR-331 | 7.033125 | 2.038362 | 5 |
| miR-335 | 1.006427 | 0.034468 | 7 | miR-335 | 0.166738 | 0.011778 | 7 |
| miR-337 | ND |  |  | miR-337 | ND |  |  |
| miR-338 | ND |  |  | miR-338 | ND |  |  |
| miR-339 | 1.029705 | 0.074005 | 8 | miR-339 | 0.155885 | 0.037134 | 7 |
| miR-340 | 0.948147 | 0.033704 | 6 | miR-340 | 4.656157 | 2.243372 | 7 |
| miR-342 | 12.36366 | 4.842359 | 8 | miR-342 | 6.643821 | 2.814147 | 8 |
| miR-345 | 1.739218 | 0.175343 | 8 | miR-345 | 1.026037 | 0.188321 | 8 |
| miR-34a | 0.980632 | 0.029579 | 6 | miR-34a | 0.141948 | 0.026599 | 8 |
| miR-34b | 3.192754 | 1.360419 | 8 | miR-34b | 2.133686 | 0.597111 | 8 |
| miR-34c | 3.127264 | 1.111165 | 8 | miR-34c | 2.684355 | 0.64027 | 8 |
| miR-361 | 1.149458 | 0.111388 | 8 | miR-361 | 1.303326 | 0.64679 | 8 |
| miR-365 | 3.612727 | 0.998509 | 8 | miR-365 | 1.416495 | 0.481006 | 8 |
| miR-367 | ND |  |  | miR-367 | ND |  |  |
| miR-368 | ND |  |  | miR-368 | ND |  |  |
| miR-369-3p | ND |  |  | miR-369-3p | ND |  |  |
| miR-369-5p | 0.973406 | 0.029483 | 7 | miR-369-5p | 0.038118 | 0.014781 | 5 |
| miR-371 | ND |  |  | miR-371 | ND |  |  |
| miR-372 | ND |  |  | miR-372 | ND |  |  |
| miR-373# | ND |  |  | miR-373# | ND |  |  |
| miR-374 | 1.955323 | 0.185445 | 8 | miR-374 | 0.403026 | 0.162793 | 4 |
| miR-375 | 22.26033 | 9.911597 | 8 | miR-375 | 24.45326 | 10.33299 | 8 |
| miR-376a | ND |  |  | miR-376a | ND |  |  |
| miR-378 | 1.173479 | 0.063316 | 8 | miR-378 | 0.360952 | 0.090577 | 8 |
| miR-379 | 0.955192 | 0.020848 | 8 | miR-379 | 0.090433 | 0.036696 | 7 |
| miR-380-3p | ND |  |  | miR-380-3p | ND |  |  |
| miR-381 | ND |  |  | miR-381 | ND |  |  |
| miR-382 | 0.957089 | 0.019462 | 7 | miR-382 | 0.22037 | 0.066108 | 8 |
| miR-383 | 1.286434 | 0.133255 | 8 | miR-383 | 0.357631 | 0.176691 | 7 |
| miR-409-5p | ND |  |  | miR-409-5p | ND |  |  |
| miR-422a | ND |  |  | miR-422a | ND |  |  |
| miR-422b | ND |  |  | miR-422b | ND |  |  |
| miR-423 | 2.475546 | 0.243494 | 8 | miR-423 | 1.364608 | 0.257038 | 8 |
| miR-424 | ND |  |  | miR-424 | ND |  |  |
| miR-425 | 2.549059 | 0.838536 | 7 | miR-425 | 1.041583 | 0.306565 | 8 |
| miR-429 | 2.151998 | 0.401249 | 8 | miR-429 | 1.892751 | 0.942768 | 4 |
| miR-432 | ND |  |  | miR-432 | ND |  |  |
| miR-433 | 1.246916 | 0.082675 | 8 | miR-433 | 0.404906 | 0.088257 | 8 |
| miR-449 | 8.117462 | 2.451482 | 8 | miR-449 | 17.35848 | 10.13508 | 8 |
| miR-450 | ND |  |  | miR-450 | ND |  |  |
| miR-451 | 2.868011 | 1.071971 | 8 | miR-451 | 2.632903 | 1.450757 | 8 |
| miR-452 | ND |  |  | miR-452 | ND |  |  |
| miR-485-3p | ND |  |  | miR-485-3p | ND |  |  |
| miR-485-5p | ND |  |  | miR-485-5p | ND |  |  |
| miR-489 | 0.819859 | 0.005464 | 4 | miR-489 | 0.058581 | 0.018743 | 4 |
| miR-490 | ND |  |  | miR-490 | ND |  |  |
| miR-494 | ND |  |  | miR-494 | ND |  |  |
| miR-497 | 1.035352 | 0.045251 | 8 | miR-497 | 0.252749 | 0.035631 | 8 |
| miR-500 | ND |  |  | miR-500 | ND |  |  |
| miR-501 | ND |  |  | miR-501 | ND |  |  |
| miR-502 | ND |  |  | miR-502 | ND |  |  |
| miR-505 | ND |  |  | miR-505 | ND |  |  |
| miR-506 | ND |  |  | miR-506 | ND |  |  |
| miR-509 | ND |  |  | miR-509 | ND |  |  |
| miR-510 | ND |  |  | miR-510 | ND |  |  |
| miR-511 | 1.002381 | 0.129721 | 4 | miR-511 | 0.07622 | 0.022102 | 6 |
| miR-513 | ND |  |  | miR-513 | ND |  |  |
| miR-514 | ND |  |  | miR-514 | ND |  |  |
| miR-515-3p | ND |  |  | miR-515-3p | ND |  |  |
| miR-515-5p | ND |  |  | miR-515-5p | ND |  |  |
| miR-517a | ND |  |  | miR-517a | ND |  |  |
| miR-517b | ND |  |  | miR-517b | ND |  |  |
| miR-517c | ND |  |  | miR-517c | ND |  |  |
| miR-518a | ND |  |  | miR-518a | ND |  |  |
| miR-518b | ND |  |  | miR-518b | ND |  |  |
| miR-518c | ND |  |  | miR-518c | ND |  |  |
| miR-518d | ND |  |  | miR-518d | ND |  |  |
| miR-518e | ND |  |  | miR-518e | ND |  |  |
| miR-519b | ND |  |  | miR-519b | ND |  |  |
| miR-519c | ND |  |  | miR-519c | ND |  |  |
| miR-519d | ND |  |  | miR-519d | ND |  |  |
| miR-519e | ND |  |  | miR-519e | ND |  |  |
| miR-520a | ND |  |  | miR-520a | ND |  |  |
| miR-520c | ND |  |  | miR-520c | ND |  |  |
| miR-520d | ND |  |  | miR-520d | ND |  |  |
| miR-520e | ND |  |  | miR-520e | ND |  |  |
| miR-520f | ND |  |  | miR-520f | ND |  |  |
| miR-520g | ND |  |  | miR-520g | ND |  |  |
| miR-520h | ND |  |  | miR-520h | ND |  |  |
| miR-521 | ND |  |  | miR-521 | ND |  |  |
| miR-522 | ND |  |  | miR-522 | ND |  |  |
| miR-7 | 0.915587 | 0.014294 | 6 | miR-7 | 1.493734 | 0.690132 | 6 |
| miR-9 | ND |  |  | miR-9 | ND |  |  |
| miR-92 | 130.0014 | 32.52349 | 8 | miR-92 | 97.13353 | 11.80491 | 8 |
| miR-93 | 9.27328 | 1.97632 | 8 | miR-93 | 7.385785 | 0.66354 | 8 |
| miR-95 | 1.051476 | 0.039776 | 8 | miR-95 | 0.88021 | 0.647417 | 8 |
| miR-96 | ND |  |  | miR-96 | ND |  |  |
| miR-98 | 1.011286 | 0.041399 | 8 | miR-98 | 0.422347 | 0.225468 | 8 |
| miR-99a | 6.970849 | 0.825762 | 8 | miR-99a | 8.118018 | 1.518184 | 8 |
| miR-99b | 3.463186 | 0.902716 | 8 | miR-99b | 1.890421 | 0.690947 | 8 |

**Table S1. Relative expression values for healthy and asthma biopsy samples.** RT-PCR was performed on RNA isolated from human airway biopsies from either healthy (non-asthmatic) or mild asthmatic patients. The expression profile of 227 miRNAs was measured (Applied Biosystems) and the ΔCT calculated based on normalisation to RNU44 (n=8). The relative expression of each was calculated (2-(ΔCT individual miRNA – mean of 227 miRNAs)). Standard error of the mean (SEM). Not detected (ND).
