# Supplementary material for: MicroRNA Expression Profiling in Mild Asthmatic Human Airways and Effect of Corticosteroid Therapy
Source: PLoS One. 2009 Jun 12;4(6):e5889. doi: 10.1371/journal.pone.0005889 (PMC2690402; doi:10.1371/journal.pone.0005889)
Supplement: Table S2 — Relative expression values for asthma biopsy samples before and after budesonide treatment. (0.42 MB DOC) [file pone.0005889.s002.doc]

Table S2.

| *Before budesonide* |  |  |  | *After budesonide* |  |  |  |
| --- | --- | --- | --- | --- | --- | --- | --- |
| *miRNA* | *2^-(Δ∆CT^)* | *SEM* | *sample n* | *miRNA* | *2^-(Δ∆CT^)* | *SEM* | *sample n* |
| let-7a | 6.46391 | 1.296374 | 5 | let-7a | 11.70637 | 2.759915 | 5 |
| let-7b | 80.57229 | 11.92984 | 5 | let-7b | 122.6658 | 17.50351 | 5 |
| let-7c | 18.60152 | 3.459467 | 5 | let-7c | 23.3686 | 5.118897 | 5 |
| let-7d | 0.845908 | 0.058233 | 5 | let-7d | 1.749143 | 0.301485 | 5 |
| let-7e | 0.064888 | 0.022092 | 3 | let-7e | 0.063233 | 0.020329 | 5 |
| let-7f | 1.114813 | 0.295972 | 5 | let-7f | 1.818733 | 0.479417 | 5 |
| let-7g | 3.617701 | 0.811637 | 5 | let-7g | 3.793912 | 0.746221 | 5 |
| miR-1 | 0.274983 | 0.099495 | 5 | miR-1 | 0.19914 | 0.073024 | 5 |
| miR-100 | 10.6168 | 2.067388 | 5 | miR-100 | 15.68326 | 2.083723 | 5 |
| miR-101 | 0.411621 | 0.090752 | 4 | miR-101 | 0.280075 | 0.087165 | 5 |
| miR-103 | 4.211859 | 0.944624 | 5 | miR-103 | 4.526291 | 0.987556 | 5 |
| miR-106b | 0.993304 | 0.21464 | 5 | miR-106b | 1.592069 | 0.438723 | 5 |
| miR-107 | 0.078503 | 0.035596 | 4 | miR-107 | 0.08894 | 0.046273 | 5 |
| miR-10a | 1.050784 | 0.384009 | 5 | miR-10a | 1.2439 | 0.498024 | 5 |
| miR-122a | ND |  |  | miR-122a | ND |  |  |
| miR-124a | ND |  |  | miR-124a | ND |  |  |
| miR-125a | 83.50903 | 18.89892 | 5 | miR-125a | 96.95527 | 25.02652 | 5 |
| miR-125b | 61.71186 | 15.36872 | 5 | miR-125b | 125.7219 | 36.82214 | 5 |
| miR-126* | 1.610766 | 0.457671 | 5 | miR-126* | 0.413456 | 0.220145 | 4 |
| miR-127 | 2.294998 | 0.988652 | 5 | miR-127 | 6.457114 | 2.4654 | 5 |
| miR-128b | ND |  |  | miR-128b | ND |  |  |
| miR-130a | 2.608266 | 0.391418 | 5 | miR-130a | 2.037544 | 0.473633 | 5 |
| miR-130b | 0.594582 | 0.075477 | 5 | miR-130b | 0.631291 | 0.118097 | 5 |
| miR-132 | 0.086904 | 0.015034 | 4 | miR-132 | 1.614099 | 0.650702 | 5 |
| miR-133a | 0.959034 | 0.636727 | 5 | miR-133a | 0.497309 | 0.227226 | 5 |
| miR-133b | 1.762459 | 0.445107 | 5 | miR-133b | 0.295196 | 0.105685 | 5 |
| miR-134 | 0.225265 | 0.081504 | 3 | miR-134 | 0.731499 | 0.307347 | 5 |
| miR-135a | 0.060166 | 0.02601 | 2 | miR-135a | 0.034987 | 0.011271 | 4 |
| miR-135b | 0.347886 | 0.126993 | 5 | miR-135b | 0.600396 | 0.0882 | 5 |
| miR-137 | ND |  |  | miR-137 | ND |  |  |
| miR-139 | 0.100224 | 0.024109 | 4 | miR-139 | 0.210035 | 0.058262 | 5 |
| miR-140 | 5.432999 | 3.567108 | 5 | miR-140 | 3.380908 | 0.670747 | 5 |
| miR-141 | 3.507583 | 0.961627 | 5 | miR-141 | 2.700723 | 0.434813 | 5 |
| miR-142-3p | 4.889026 | 1.37625 | 5 | miR-142-3p | 12.62013 | 3.098161 | 5 |
| miR-142-5p | 0.620415 | 0.341747 | 5 | miR-142-5p | 0.927865 | 0.23536 | 5 |
| miR-143 | 0.465437 | 0.164694 | 4 | miR-143 | 0.983405 | 0.230655 | 5 |
| miR-145 | 12.79346 | 3.171164 | 5 | miR-145 | 17.52776 | 5.094003 | 5 |
| miR-146a | 5.08498 | 1.200688 | 5 | miR-146a | 4.980498 | 1.380994 | 5 |
| miR-146b | 7.833099 | 2.238234 | 5 | miR-146b | 6.931614 | 2.788812 | 5 |
| miR-147 | ND |  |  | miR-147 | ND |  |  |
| miR-148a | 1.75097 | 0.447564 | 5 | miR-148a | 1.094809 | 0.278371 | 5 |
| miR-148b | 0.201089 | 0.044379 | 5 | miR-148b | 0.115835 | 0.026271 | 5 |
| miR-149 | 1.702902 | 0.449066 | 5 | miR-149 | 2.39309 | 0.527858 | 5 |
| miR-151 | 2.100501 | 0.44015 | 3 | miR-151 | 1.784713 | 0.338136 | 5 |
| miR-152 | 1.898678 | 0.288632 | 5 | miR-152 | 3.448958 | 1.056896 | 5 |
| miR-153 | ND |  |  | miR-153 | ND |  |  |
| miR-155 | 2.209223 | 0.4917 | 4 | miR-155 | 3.113434 | 1.151318 | 5 |
| miR-15a | 0.072382 | 0.033103 | 5 | miR-15a | 0.098441 | 0.035274 | 5 |
| miR-15b | 6.564372 | 1.514618 | 5 | miR-15b | 8.639443 | 1.614834 | 5 |
| miR-16 | 119.7026 | 33.79496 | 5 | miR-16 | 130.4547 | 41.52521 | 5 |
| miR-17-3p | ND |  |  | miR-17-3p | ND |  |  |
| miR-17-5p | 1.188306 | 0.372438 | 5 | miR-17-5p | 1.027842 | 0.153228 | 5 |
| miR-181b | 5.772876 | 0.636445 | 5 | miR-181b | 12.01937 | 2.892896 | 5 |
| miR-181c | 0.020855 | 0.002579 | 3 | miR-181c | 0.096545 | 0.045928 | 3 |
| miR-181d | 2.250058 | 0.606544 | 5 | miR-181d | 5.317487 | 1.556913 | 5 |
| miR-182 | 1.247244 | 0.135776 | 5 | miR-182 | 0.478969 | 0.134824 | 5 |
| miR-183 | 0.108385 | 0.040897 | 4 | miR-183 | 0.076855 | 0.016688 | 4 |
| miR-184 | ND |  |  | miR-184 | ND |  |  |
| miR-186 | 2.277975 | 0.654596 | 5 | miR-186 | 1.256994 | 0.393818 | 5 |
| miR-187 | 0.758622 | 0.392359 | 4 | miR-187 | 1.124528 | 0.52744 | 5 |
| miR-18a | 0.035072 | 0.010184 | 5 | miR-18a | 0.021374 | 0.009941 | 5 |
| miR-190 | ND |  |  | miR-190 | ND |  |  |
| miR-191 | 32.91345 | 5.132442 | 5 | miR-191 | 31.98195 | 3.759323 | 5 |
| miR-192 | 0.288212 | 0.070429 | 5 | miR-192 | 0.301354 | 0.062582 | 5 |
| miR-193a | 0.19448 | 0.060042 | 4 | miR-193a | 0.131456 | 0.032475 | 5 |
| miR-193b | 0.129011 | 0.057493 | 3 | miR-193b | 0.291647 | 0.100293 | 5 |
| miR-194 | 0.210878 | 0.047897 | 5 | miR-194 | 0.257197 | 0.036635 | 5 |
| miR-195 | 4.374485 | 1.129324 | 5 | miR-195 | 2.182094 | 0.395783 | 5 |
| miR-196a | ND |  |  | miR-196a | ND |  |  |
| miR-196b | 3.332547 | 2.022079 | 3 | miR-196b | 0.022314 | 0.004951 | 5 |
| miR-197 | 5.947574 | 1.2853 | 5 | miR-197 | 6.657325 | 0.618527 | 5 |
| miR-198 | ND |  |  | miR-198 | ND |  |  |
| miR-199a | 0.116586 | 0.047212 | 3 | miR-199a | 0.428324 | 0.177083 | 5 |
| miR-199b | 0.116278 | 0.035248 | 4 | miR-199b | 0.410394 | 0.149471 | 5 |
| miR-19a | 0.713614 | 0.239371 | 5 | miR-19a | 0.80624 | 0.245436 | 5 |
| miR-19b | 7.523485 | 1.354736 | 5 | miR-19b | 12.21529 | 2.595839 | 5 |
| miR-200a | ND |  |  | miR-200a | ND |  |  |
| miR-200c | 237.3176 | 88.09194 | 5 | miR-200c | 220.3274 | 13.35709 | 5 |
| miR-203 | 2.980415 | 1.566766 | 5 | miR-203 | 1.339385 | 0.132799 | 5 |
| miR-204 | 0.628882 | 0.111967 | 5 | miR-204 | 0.292033 | 0.025751 | 5 |
| miR-205 | 8.342377 | 3.347153 | 3 | miR-205 | 21.73201 | 6.336771 | 5 |
| miR-206 | 0.156586 | 0.12265 | 3 | miR-206 | 0.025493 | 0.010094 | 3 |
| miR-208 | ND |  |  | miR-208 | ND |  |  |
| miR-20a | 5.932907 | 0.911743 | 5 | miR-20a | 10.86952 | 1.549152 | 5 |
| miR-20b | 0.279813 | 0.057347 | 4 | miR-20b | 14.2043 | 13.66437 | 5 |
| miR-21 | 3.379594 | 0.722379 | 5 | miR-21 | 18.31867 | 4.053626 | 5 |
| miR-210 | 1.261008 | 0.305004 | 5 | miR-210 | 1.713279 | 0.33595 | 5 |
| miR-211 | ND |  |  | miR-211 | ND |  |  |
| miR-213 | 11.54792 | 7.529042 | 5 | miR-213 | 0.143796 | 0.05279 | 5 |
| miR-214 | 11.10516 | 5.598909 | 3 | miR-214 | 45.60053 | 16.86599 | 5 |
| miR-215 | ND |  |  | miR-215 | ND |  |  |
| miR-216 | ND |  |  | miR-216 | ND |  |  |
| miR-217 | ND |  |  | miR-217 | ND |  |  |
| miR-218 | 4.94563 | 4.386249 | 5 | miR-218 | 1.321427 | 0.308699 | 5 |
| miR-219 | ND |  |  | miR-219 | ND |  |  |
| miR-22 | ND |  |  | miR-22 | ND |  |  |
| miR-220 | ND |  |  | miR-220 | ND |  |  |
| miR-220 | ND |  |  | miR-220 | ND |  |  |
| miR-221 | 0.194863 | 0.090638 | 5 | miR-221 | 1.826211 | 0.646325 | 5 |
| miR-222 | 18.88382 | 3.283081 | 5 | miR-222 | 22.19471 | 3.914821 | 5 |
| miR-223 | 107.3704 | 71.81721 | 5 | miR-223 | 69.77399 | 18.15416 | 5 |
| miR-224 | 0.137716 | 0.048848 | 4 | miR-224 | 0.234965 | 0.02277 | 5 |
| miR-23a | 0.631914 | 0.141817 | 5 | miR-23a | 0.318488 | 0.185352 | 4 |
| miR-23b | 1.00273 | 0.31 | 5 | miR-23b | 3.060253 | 0.615077 | 5 |
| miR-24 | 48.47147 | 9.595456 | 5 | miR-24 | 59.10091 | 7.632308 | 5 |
| miR-25 | 2.709708 | 0.681932 | 5 | miR-25 | 3.378492 | 0.84561 | 5 |
| miR-26a | 141.7872 | 28.18907 | 5 | miR-26a | 169.3185 | 33.12824 | 5 |
| miR-26b | 10.26726 | 2.343521 | 5 | miR-26b | 13.80334 | 1.31921 | 5 |
| miR-27a | 5.935564 | 1.609133 | 5 | miR-27a | 9.793865 | 1.910969 | 5 |
| miR-27b | 1.844297 | 0.451978 | 5 | miR-27b | 5.62158 | 1.352053 | 5 |
| miR-28 | 0.432803 | 0.20135 | 5 | miR-28 | 0.952734 | 0.239071 | 5 |
| miR-296 | 0.419008 | 0.116585 | 5 | miR-296 | 0.492818 | 0.296512 | 4 |
| miR-299-5p | 0.046318 | 0.019978 | 3 | miR-299-5p | 0.102179 | 0.042546 | 4 |
| miR-29a | 6.121253 | 1.46513 | 5 | miR-29a | 4.839842 | 1.018634 | 5 |
| miR-29c | 1.680479 | 0.663963 | 5 | miR-29c | 1.236263 | 0.464197 | 5 |
| miR-301 | 0.26588 | 0.057029 | 5 | miR-301 | 0.293411 | 0.101303 | 5 |
| miR-302a | ND |  |  | miR-302a | ND |  |  |
| miR-302c | ND |  |  | miR-302c | ND |  |  |
| miR-302d | ND |  |  | miR-302d | ND |  |  |
| miR-30a-3p | 2.443306 | 0.341422 | 5 | miR-30a-3p | 2.614141 | 0.47409 | 5 |
| miR-30a-5p | 14.97933 | 2.902953 | 5 | miR-30a-5p | 36.85443 | 7.561671 | 5 |
| miR-30b | 25.13326 | 7.826434 | 5 | miR-30b | 9.581658 | 2.756736 | 5 |
| miR-30c | 19.24114 | 3.151219 | 5 | miR-30c | 13.70348 | 3.457125 | 5 |
| miR-30d | 11.78594 | 2.875825 | 5 | miR-30d | 6.985863 | 1.407784 | 5 |
| miR-30e-3p | 2.461054 | 0.342242 | 5 | miR-30e-3p | 1.444699 | 0.209509 | 5 |
| miR-30e-5p | 1.496602 | 0.418531 | 5 | miR-30e-5p | 2.03743 | 0.40714 | 5 |
| miR-31 | 4.837836 | 0.927264 | 5 | miR-31 | 7.352684 | 0.848683 | 5 |
| miR-32 | 0.046542 | 0.019747 | 3 | miR-32 | 0.053792 | 0.017837 | 5 |
| miR-320 | 20.99495 | 6.832896 | 3 | miR-320 | 10.80667 | 2.785581 | 5 |
| miR-323 | ND |  |  | miR-323 | ND |  |  |
| miR-324-3p | 0.579329 | 0.226018 | 5 | miR-324-3p | 1.667181 | 0.493952 | 5 |
| miR-324-5p | 0.236819 | 0.074863 | 5 | miR-324-5p | 0.891085 | 0.239654 | 5 |
| miR-325 | ND |  |  | miR-325 | ND |  |  |
| miR-326 | 2.911965 | 1.969789 | 3 | miR-326 | 0.044297 | 0.010195 | 4 |
| miR-328 | 4.990092 | 1.577526 | 5 | miR-328 | 5.652453 | 0.764317 | 5 |
| miR-33 | ND |  |  | miR-33 | ND |  |  |
| miR-330 | 0.083387 | 0.017174 | 4 | miR-330 | 0.066669 | 0.010304 | 5 |
| miR-331 | 6.835337 | 2.913695 | 3 | miR-331 | 10.35527 | 2.250256 | 5 |
| miR-335 | 0.156944 | 0.009663 | 5 | miR-335 | 0.211172 | 0.050552 | 5 |
| miR-337 | ND |  |  | miR-337 | ND |  |  |
| miR-338 | ND |  |  | miR-338 | ND |  |  |
| miR-339 | 0.157736 | 0.041188 | 5 | miR-339 | 0.278929 | 0.073212 | 5 |
| miR-340 | 4.81945 | 2.945299 | 5 | miR-340 | 0.110624 | 0.022762 | 5 |
| miR-342 | 8.475625 | 4.466585 | 5 | miR-342 | 18.60465 | 5.292569 | 5 |
| miR-345 | 1.292402 | 0.239947 | 5 | miR-345 | 1.165754 | 0.286068 | 5 |
| miR-34a | 0.140921 | 0.034084 | 5 | miR-34a | 0.216702 | 0.041692 | 5 |
| miR-34b | 1.933461 | 0.762448 | 5 | miR-34b | 2.036455 | 0.385587 | 5 |
| miR-34c | 2.622564 | 0.849856 | 5 | miR-34c | 2.780364 | 0.598118 | 5 |
| miR-361 | 1.670476 | 0.999525 | 5 | miR-361 | 0.853547 | 0.242533 | 5 |
| miR-365 | 1.921287 | 0.74755 | 5 | miR-365 | 4.374228 | 1.10346 | 5 |
| miR-367 | ND |  |  | miR-367 | ND |  |  |
| miR-368 | ND |  |  | miR-368 | ND |  |  |
| miR-369-3p | ND |  |  | miR-369-3p | ND |  |  |
| miR-369-5p | ND |  |  | miR-369-5p | ND |  |  |
| miR-371 | ND |  |  | miR-371 | ND |  |  |
| miR-372 | ND |  |  | miR-372 | ND |  |  |
| miR-373# | ND |  |  | miR-373# | ND |  |  |
| miR-374 | 0.410668 | 0.208153 | 3 | miR-374 | 0.386797 | 0.099166 | 5 |
| miR-375 | 33.45687 | 15.27255 | 5 | miR-375 | 9.584979 | 1.445746 | 5 |
| miR-376a | 0.062584 | 0.012701 | 4 | miR-376a | 0.094731 | 0.029726 | 5 |
| miR-378 | 0.342459 | 0.097789 | 5 | miR-378 | 0.531769 | 0.130128 | 5 |
| miR-379 | 0.10186 | 0.055368 | 5 | miR-379 | 0.137733 | 0.074225 | 5 |
| miR-380-3p | ND |  |  | miR-380-3p | ND |  |  |
| miR-381 | ND |  |  | miR-381 | ND |  |  |
| miR-382 | 0.203485 | 0.073696 | 5 | miR-382 | 0.881358 | 0.44896 | 5 |
| miR-383 | 0.406903 | 0.266962 | 5 | miR-383 | 0.063724 | 0.012094 | 5 |
| miR-409-5p | ND |  |  | miR-409-5p | ND |  |  |
| miR-422a | ND |  |  | miR-422a | ND |  |  |
| miR-422b | 0.486878 | 0.294664 | 5 | miR-422b | 0.026259 | 0.006363 | 4 |
| miR-423 | 1.403445 | 0.361091 | 5 | miR-423 | 3.963026 | 0.87129 | 5 |
| miR-424 | ND |  |  | miR-424 | ND |  |  |
| miR-425 | 0.72477 | 0.203846 | 5 | miR-425 | 0.611344 | 0.163589 | 5 |
| miR-429 | 2.380083 | 1.021588 | 3 | miR-429 | 0.819994 | 0.159301 | 5 |
| miR-432 | ND |  |  | miR-432 | ND |  |  |
| miR-433 | 0.471617 | 0.115555 | 5 | miR-433 | 1.147535 | 0.299494 | 5 |
| miR-449 | 7.521525 | 3.450955 | 5 | miR-449 | 62.34093 | 17.13129 | 5 |
| miR-450 | ND |  |  | miR-450 | ND |  |  |
| miR-451 | 2.878697 | 2.044288 | 5 | miR-451 | 3.688698 | 2.347803 | 5 |
| miR-452 | 0.02583 | 0.001861 | 3 | miR-452 | 0.016081 | 0.007143 | 4 |
| miR-485-3p | ND |  |  | miR-485-3p | ND |  |  |
| miR-485-5p | ND |  |  | miR-485-5p | ND |  |  |
| miR-489 | 0.065099 | 0.026941 | 3 | miR-489 | 0.015985 | 0.003456 | 3 |
| miR-490 | ND |  |  | miR-490 | ND |  |  |
| miR-494 | ND |  |  | miR-494 | ND |  |  |
| miR-497 | 0.289792 | 0.048303 | 5 | miR-497 | 0.344406 | 0.074923 | 5 |
| miR-500 | ND |  |  | miR-500 | ND |  |  |
| miR-501 | ND |  |  | miR-501 | ND |  |  |
| miR-502 | ND |  |  | miR-502 | ND |  |  |
| miR-505 | ND |  |  | miR-505 | ND |  |  |
| miR-506 | ND |  |  | miR-506 | ND |  |  |
| miR-509 | ND |  |  | miR-509 | ND |  |  |
| miR-510 | ND |  |  | miR-510 | ND |  |  |
| miR-511 | 0.055868 | 0.009385 | 5 | miR-511 | 0.089804 | 0.026811 | 5 |
| miR-513 | ND |  |  | miR-513 | ND |  |  |
| miR-514 | ND |  |  | miR-514 | ND |  |  |
| miR-515-3p | ND |  |  | miR-515-3p | ND |  |  |
| miR-515-5p | ND |  |  | miR-515-5p | ND |  |  |
| miR-517a | ND |  |  | miR-517a | ND |  |  |
| miR-517b | ND |  |  | miR-517b | ND |  |  |
| miR-517c | ND |  |  | miR-517c | ND |  |  |
| miR-518a | ND |  |  | miR-518a | ND |  |  |
| miR-518b | ND |  |  | miR-518b | ND |  |  |
| miR-518c | ND |  |  | miR-518c | ND |  |  |
| miR-518d | ND |  |  | miR-518d | ND |  |  |
| miR-518e | ND |  |  | miR-518e | ND |  |  |
| miR-519b | ND |  |  | miR-519b | ND |  |  |
| miR-519c | ND |  |  | miR-519c | ND |  |  |
| miR-519d | ND |  |  | miR-519d | ND |  |  |
| miR-519e | ND |  |  | miR-519e | ND |  |  |
| miR-520a | ND |  |  | miR-520a | ND |  |  |
| miR-520c | ND |  |  | miR-520c | ND |  |  |
| miR-520d | ND |  |  | miR-520d | ND |  |  |
| miR-520e | ND |  |  | miR-520e | ND |  |  |
| miR-520f | ND |  |  | miR-520f | ND |  |  |
| miR-520g | ND |  |  | miR-520g | ND |  |  |
| miR-520h | ND |  |  | miR-520h | ND |  |  |
| miR-521 | ND |  |  | miR-521 | ND |  |  |
| miR-522 | ND |  |  | miR-522 | ND |  |  |
| miR-7 | 1.421823 | 0.854276 | 4 | miR-7 | 0.055657 | 0.015156 | 4 |
| miR-9 | 0.019722 | 0.008783 | 3 | miR-9 | 0.015777 | 0.002791 | 4 |
| miR-92 | 93.78332 | 7.571548 | 5 | miR-92 | 151.2459 | 18.10777 | 5 |
| miR-93 | 8.369234 | 0.664488 | 5 | miR-93 | 13.56698 | 2.588046 | 5 |
| miR-95 | 0.211367 | 0.034309 | 5 | miR-95 | 0.266998 | 0.034904 | 5 |
| miR-96 | ND |  |  | miR-96 | ND |  |  |
| miR-98 | 0.620322 | 0.35453 | 5 | miR-98 | 0.173739 | 0.040783 | 5 |
| miR-99a | 10.15019 | 1.294989 | 5 | miR-99a | 10.15792 | 1.390373 | 5 |
| miR-99b | 1.932103 | 1.058034 | 5 | miR-99b | 1.165443 | 0.617719 | 5 |

**Table S2. Relative expression values for asthma biopsy samples before and after budesonide treatment.** RT-PCR was performed on RNA isolated from human airway biopsies from mild asthmatic patients before or after budesonide treatment. The expression profile of 227 miRNAs was measured (Applied Biosystems) and the average ΔCT calculated based on normalisation to RNU44 (n=5). The relative expression of each was calculated (2-(ΔCT individual miRNA – mean of 227 miRNAs)). Standard error of the mean (SEM). Not detected (ND).
