# Supplementary material for: MicroRNA Expression Profiling in Mild Asthmatic Human Airways and Effect of Corticosteroid Therapy
Source: PLoS One. 2009 Jun 12;4(6):e5889. doi: 10.1371/journal.pone.0005889 (PMC2690402; doi:10.1371/journal.pone.0005889)
Supplement: Table S3 — Bioinformatic analysis of highly expressed miRNAs expressed in airway biopsies. (0.05 MB DOC) [file pone.0005889.s003.doc]

Table S3.

| microRNA | sequence/  genomic location/  target frequency | Selected targets | TF binding sites |
| --- | --- | --- | --- |
| miR-92 | uauugcacuugucccggccugu  chromosome 13  567 targets | CD69, FLJ20273, TGAV, MYO1B, CDH11X, MAP2K4, AP2K4, NFIA, CPEB2, COL5A1, MPR2, COL1A2, IF4G2, SMAD7, RAF3, HAND1, EDG1, HAND2, GATA6, GATA2, PAX3, SOCS5, SOCS6, E2F3, NFAT5, SMAD6 | p53, CHOP, TGIF,  OCT1, MYCMAX, ZID, PAX5, TAXCREB, AP2,  POU3F2, CMYB,  NFkBp65, STAT5A,  STAT5B, NFkB,  CREL, NKX61 |
| miR-26a  miR-26b | uucaaguaauccaggauaggcu  chromosome 3  601 targets  uucaaguaauucaggauaggu  chromosome 2 | PTEN, SMAD1, MAPK6,  ATF2, SMAD1, AP3K2, EIF4G2, EIF5, EIF3S10, COL1A2, CD200, ITGB8 | USF, NMYC, MYCMAX, ARNT  SRF, AHR, OLF1, NFKBp50, STAT, AHRARNT, MYOGNF1, P300, ZID |
| miR-16 | uagcagcacguaaauauuggcg  chromosome 13  733 targets | FGF2, COL12A1, FGF7,  CD28, FGFR1, SMAD7,  E2F7, SMAD5, GHR,  MAP2K1, SMAD3, COL24A1, PDCD4, SOCS6, E2F3, ELL | E4BP4, FOXJ2, PBX1, NHF1, NKX61, AREB6, TST1, CMYB, PBX1, CHOP, IRF1 |
| let-7a  let-7b  let-7c | ugagguaguagguuguauaguu  chromosome 9  616 targets  ugagguaguagguugugugguu  chromosome 22  ugagguaguagguuguaugguu  chromosome 21 | HMGA2, IGF2BP1, MASP1, TGFBR1, IGF1R, GDF6,  COL1A2, ADAMTS8, COL3A1, STX3, MAPK6,  CASP3, IGF2BP3, IGF2BP2, COL14A1, DMD, LIN28, EIF4G2 | NKX61  COUP, PPARG  POU3F2, OCT1, FREAC3, HNF1, FOXJ2, CDP, POU6F1, FREAC4,  FREAC2, CART1, NKX22, |
| miR-125a  miR-125b | ucccugagacccuuuaaccuguga  chromosome 19  488 targets  ucccugagacccuaacuuguga  chromosome 11 | STARD13, IRF4, IER3IP1, BAK1, TNFSF4, SOX11, TRIAP1, BMF, IL16, MAP3K10 | ROAZ, RFX1, ARP1, NRSF, PAX4, PAX2, PPARG, ZID,  CMYB, MYB, YY1, GATA1, FOXO1, CDP, SEF1, CHX10, PAX4, ROAZ |
| miR-220c | uaauacugccggguaaugaugga  chromosome 12  602 targets | ZEB2, LEPR, MAP2, CCNJ, TFAP2A, CFL2, MMD, THRAP1, FN1, EPS8, NTF3, NFIB, NRIP1, NCOA2, MAP4K4, MAP4K3, E2F3, COL4A3, FNDC3B, PCDH19, EIF2S1, VEGFA, MMD2 | AREB6, HMX1, TAL1BETAE47 |
| miR-320 | aaaagcuggguugagagggcga  chromosome 8  482 targets | YOD1, CREB5, BMPR1A, PCDHAC1, PCDHAC2, PCDHA1, PCDHA13, E2F7, PCDH19, RAB14, TFAP2B, EIF3S1, ELL2, ITGB1, IGF2BP3, EREG | NMYC, USF,  HOXA9, TAL1BETAE47, TAL1ALPHAE47, RFX1 |
| miR-24 | uggcucaguucagcaggaacag  chromosome 9  349 targets | CALCR, KCNK2, BCL2L11, SNN, TOP1, ELL, KCTD21, EDG1, TRAF7, RAP1B, TNFRSF19, PLOD2, TCF7, MAG1 | RFX1, GFI1 |
| miR-223 | ugucaguuugucaaauacccca  chromosome x  172 targets | FBXW7, MYO5B, RHOB, SP3, CALML4, HSP90B1, NFIA, NFIB,  IGF1R, ALCAM, ATBF1, FGFR2, CBFB | CEBP, CEBPB, POU3F2 |
| miR-30a  miR-30b  miR-30c | uguaaacauccucgacuggaag  chromosome 6  873 targets  uguaaacauccuacacucagcu  chromosome 8  uguaaacauccuacacucucagc  chromosome 1 | CELSR3, EED, NFAT5, EEA1, FNDC3A, NFIB, NRIP1, KLF9, CACNB2, ADAM19, ADAMTS3, FAP, TFDP1, E2F7, SOCS3, SP4, ELL2, PCDH17, ITGA6, MAP4K4, CASP3, COL13A1, PCDH10, P4HA2, IL1A, DOCK4, SOCS1, PCDH20, EDNRA, SMAD1, IRF4, IGF2R, SOCS6 | NFAT, AP1, FREAC4  HFH1  ISRE, FREAC2, GRE, HOX13, HOXA9, LUN1, FOXO1, FOXO3, FOXO4 |
| miR-191 | caacggaaucccaaaagcagcug  chromosome 3  32 targets | TMOD2, TAF5, TJP1, CEBPB, EGR1, MAP3K12, NRCAM | HEN1, TAL1BETAE47, TAL1ALPHAE47, CMYB, AREB6, LMO2COM, MYOD, AHRARNT, IRF7, CEBPA, RORA2 |
| miR-375 | uuuguucguucggcucgcguga  chromosome 2  211 targets | SLC16A2, USP6, EIF4G3, RASD1, USP32, JAK2 | PPARG, AREB6,  CP2 |
| miR-222 | agcuacaucuggcuacugggu  chromosome x  miR-251 | TCF12, MARK1, EIF5A2, CREBZF, NRK, BCL2L11, BMF, IRF2, PCDHAC1, PCDHA1, PCDHA13 | CDC5, NKX25, PAX3, SRF |
| miR-342 | aggggugcuaucugugauuga  chromosome 14  163 targets | JMJD3, TFDP2, EDA, LPPR4 | ARP1, NFY, NCX, P300, LUN1 |
| miR-214 | acagcaggcacagacaggcagu  chromosome 1  375 targets | NARG1, SDAD1, MAPK1, KRTAP4-4, NOMO1, NOMO2, NOMO3, CSF1, FNDC5,  TFAP2C | ZIC1, ZIC2, ZIC3, GATA1, TCF11MAFG, NFE2 |

**Table S3. Bioinformatic analysis of highly expressed miRNAs expressed in airway biopsies.** Those miRNAs that were highly expressed in airway biopsies were subjected to bioinformatic analysis. The miRBase microRNA registry ([http://microrna.sanger.ac.uk](http://microrna.sanger.ac.uk/)) was initially used to identify individual miRNAs and obtain the chromosomal location, as well as providing a link to target prediction software. The public target prediction database TargetScan 4.0 ([http://www.targetscan.org](http://www.targetscan.org/)) was used to perform a search on human miRNA target sequences. The number of targets predicted by TargetScan and example targets (based on transcription factors, inflammatory genes and lung specific targets) were identified. Transcription factor binding sites were identified using Genome Browser (University of California Santa Cruz, [http://genome.ucsc.edu](http://genome.ucsc.edu/)).
